# Augmentation of donor-derived Tax-specific CTL responses by a novel Tax epitope-specific CD4+ helper T-cells in ATL patients after allogeneic hematopoietic stem cell transplantation

**DOI:** 10.1186/1742-4690-11-S1-O35

**Published:** 2014-01-07

**Authors:** Atsuhiko Hasegawa, Yotaro Tamai, Ayako Takamori, Amane Sasada, Ryuji Tanosaki, Ilseung Choi, Atae Utsunomiya, Yasuhiro Maeda, Yoshihisa Yamano, Tetsuya Eto, Ki-Ryang Koh, Hirohisa Nakamae, Youko Suehiro, Koji Kato, Shigeki Takemoto, Jun Okamura, Naokuni Uike, Mari Kannagi

**Affiliations:** 1 Department of Immunotherapeutics, Tokyo Medical and Dental University, Tokyo, Japan; 2 Clinical Laboratories Division, National Cancer Center Hospital, Tokyo, Japan; 3 Department of Hematology, National Kyushu Cancer Center, Fukuoka, Japan; 4 Department of Hematology, Imamura Bun-in Hospital, Kagoshima, Japan; 5 Division of Hematology, Department of Internal Medicine, Kinki University School of Medicine, Osaka, Japan; 6 Department of Rare Diseases Research, Institute of Medical Science, St. Marianna University Graduate School of Medicine, Kawasaki, Japan; 7 Department of Hematology, Hamanomachi Hospital, Fukuoka, Japan; 8 Department of Hematology, Graduate School of Medicine, Osaka City University, Osaka, Japan; 9 Department of Medicine and Biosystemic Science, Kyushu University Graduate School of Medical Sciences, Fukuoka, Japan; 10 Department of Hematology, National Hospital Organization Kumamoto Medical Center, Kumamoto, Japan; 11 Institute for Clinical Research, National Kyushu Cancer Center, Fukuoka, Japan

## 

Adult T-cell leukemia/ lymphoma (ATL) is an aggressive T-cell malignancy caused by human T-cell leukemia virus type 1 (HTLV-1) and characterized by extremely poor prognosis because of intrinsic drug resistance to cytotoxic agents. Recently, allogeneic hematopoietic stem cell transplantation (allo-HSCT) has been shown to be an effective treatment for ATL due to graft-versus-leukemia effects. However, donor-derived HTLV-1-specific T-cell responses in ATL patients after allo-HSCT are not well understood. In this study, blood samples from 15 ATL patients at 180 days following allo-HSCT from seronegative donors were screened for IFN-g production of donor-derived T-cells against Tax protein. Among 15 patients, Tax-specific T-cell responses were observed in 11 patients. Direct detection with Tax/MHC class I tetramers revealed that donor-derived Tax-specific CD8^+^ cytotoxic T-lymphocytes (CTLs) were present in 12 of 15 patients. We then identified a novel Tax epitope (Tax155-167) presented by HLA-DR1 (HLA-DRB1*0101) and examined the presence of Tax-specific CD4^+^ T-cells in 3 HLA-DR1^+^ patients after allo-HSCT using a newly generated Tax155-167/HLA-DR1 tetramer. Tax155-167-specific CD4^+^ T-cells were found to be present in all HLA-DR1^+^ patients tested. Furthermore, in vitro stimulation of PBMCs from HLA-DR1^+^ HLA-A*2402^+^ patients with both Tax155-167 and HLA-A*2402-restricted CTL epitope (Tax301-309) led to robust expansion of Tax301-309-specific CTLs. Our results suggest that donor-derived HTLV-1-specific both CD4^+^ and CD8^+^ T-cells could be induced in ATL patients after allo-HSCT from seronegative donors, probably due to exposure to HTLV-1 antigens from recipient-derived ATL cells, and the HTLV-1-specific CD4^+^ T-cells may contribute to efficient induction of donor-derived HTLV-1-specific CTL responses.

